# Artificial intelligence and social media on academic performance and mental well-being: Student perceptions of positive impact in the age of smart learning

**DOI:** 10.1016/j.heliyon.2024.e29523

**Published:** 2024-04-15

**Authors:** Muhammad Farrukh Shahzad, Shuo Xu, Weng Marc Lim, Xingbing Yang, Qasim Raza Khan

**Affiliations:** a College of Economics and Management, Beijing University of Technology, Beijing, PR China; b Sunway Business School, Sunway University, Sunway City, Selangor, Malaysia; c School of Business, Law and Entrepreneurship, Swinburne University of Technology, Hawthorn, Victoria, Australia; d Design and Arts, Swinburne University of Technology, Kuching, Sarawak, Malaysia; e Beijing Yuchehang Information Technology Co., Ltd, Beijing, 100089, PR China; f Department of Management Sciences, COMSATS University Islamabad, Lahore Campus, Pakistan

**Keywords:** Academic performance, Artificial intelligence, Mental well-being, Smart learning, Social media

## Abstract

The advancement of artificial intelligence (AI) and the ubiquity of social media have become transformative agents in contemporary educational ecosystems. The spotlight of this inquiry focuses on the nexus between AI and social media usage in relation to academic performance and mental well-being, and the role of smart learning in facilitating these relationships. Using partial least squares–structural equation modeling (PLS-SEM) on a sample of 401 Chinese university students. The study results reveal that both AI and social media have a positive impact on academic performance and mental well-being among university students. Furthermore, smart learning serves as a positive mediating variable, amplifying the beneficial effects of AI and social media on both academic performance and mental well-being. These revelations contribute to the discourse on technology-enhanced education, showing that embracing AI and social media can have a positive impact on student performance and well-being.

## 1 Introduction

In recent times, the penetration of artificial intelligence (AI) and the ubiquity of social media have significantly transformed our daily lives, including how we communicate, interact, and access information [1]. This transformation holds particular salience for the younger generation, for whom the integration of AI and social media has become nothing short of indispensable in both their educational and daily experiences. Despite the essential nature of these technologies, their synergistic impact on academic performance and mental well-being remains underexplored and thus necessitates research attention [2].

Emerging as a compelling frontier in AI applications, Generative AI, typified by ChatGPT, have emerged as an influential virtual assistant in higher education [3]. These AI systems offer multifaceted advantages, rendering a critical assessment of their impact on both the academic performance and mental well-being of university students' imperative [4].

AI and social media have not only revolutionized educational paradigms but also recast the modes of student engagement with digital content and their social environment [5]. Past research underscores the ease with which AI enables information accessibility, peer connectivity, and participation in digital communities [6]. Yet, the unrelenting exposure to algorithmically-curated content and the seductive appeal of social platforms may have consequential implications for both academic performance and mental well-being [7].

Previous investigations revealed that the incorporation of Generative AI, such as ChatGPT [3], into university education can engender enhanced academic outcomes [8]. By furnishing personalized assistance, real-time feedback, and a wealth of information, these AI architectures empower students to decode complex subject matter, sharpen problem-solving acumen, and elevate their learning journeys [9].

Moreover, AI technologies serve as robust tools for effective time management, task prioritization, and as repositories of supplemental educational resources [10]. While previous studies have interrogated the role of AI and social media on academic performance, they have often fallen short of a comprehensive analysis [1,11,12]. Therefore, our inquiry focuses on student perceptions of AI technology that can assist in education, like ChatGPT, to enhance learning efficacy through tailored educational experiences, adaptive assessments, and intelligent tutoring systems.

University life frequently comes laden with anxiety, pressure, and stress—emotional states that AI could either aggravate or ameliorate [13,14]. The nebulous impact of social media on the mental well-being of young individuals also remains a contested domain in the academic discourse [15,16]. Our study pivots to accentuate student perceptions of the positive influence of social media on academic performance, countering the common narrative of its detrimental psychological impact [17]. Prior research has explored the addictive tendencies of social media and the corresponding threats to mental well-being, such as cyberbullying and online harassment [[18], [19], [20]]. Accordingly, a nuanced understanding of the impacts of these technologies on academic performance and mental health is warranted.

To this end, we adopt social learning theory to explore smart learning, an educational approach buttressed by smart technologies such as AI and social media, to unravel the complex dynamics at play [21]. This enables us to enlighten young students on the strategic deployment of AI and social media to bolster both their academic endeavors and mental wellness. In doing so, we construct a navigational blueprint for the youth to capitalize on smart learning methodologies for holistic self-improvement. This is consistent with prior studies that have endorsed the positive influence of Internet use on student academic performance [22].

Corroborating our arguments, extant literature provides policy directives aimed at fostering a balanced digital ecosystem to buttress academic success and emotional well-being [23]. Despite the growing adoption of AI tools like ChatGPT and social media like Facebook, WeChat, and WhatsApp, a glaring research lacuna exists [[24], [25], [26]]. The specific impact of AI, especially in the transformative age of Generative AI [3] on both academic and emotional well-being remains under-explored, while the mediating role of smart learning in the nexus between AI, social media, and user experiences remains under-researched [27,28].

To address these research gaps, we aim to explore how students view AI and social media's influence on academic performance and mental wellness through smart learning. Our investigation is guided by two research questions: First, how do students believe AI and social media correlate with academic performance and mental well-being? Second, does smart learning function as a mediating variable in how students perceive the relationship between AI, social media, academic performance, and mental well-being?

The contributions of this study are twofold, aiming to advance both theoretical understanding and practical applications. From a theoretical standpoint, this research provides a comprehensive analysis of the synergistic relationship between AI technologies, specifically Generative AI like ChatGPT [29], and social media platforms in the context of academic performance and mental well-being. By adopting social learning theory as a foundational lens, we introduce smart learning as a mediating variable—an area hitherto under-researched. This theoretical enrichment not only fills existing gaps in the literature but also lays the groundwork for future studies examining the complex interplay of these digital technologies within educational and psychological frameworks.

On the practical front, this study offers actionable insights for educators, administrators, and policy-makers. The empirical findings will equip academic institutions with the tools to incorporate AI and social media more effectively in curricula, thereby enhancing educational outcomes. Moreover, this study serves as a guide for students, enlightening them on how to strategically deploy AI and social media for academic success and improved mental wellness. Thus, our study not only deepens the academic discourse but also provides a pragmatic roadmap for stakeholders interested in leveraging digital technologies for holistic educational and psychological betterment.

## 2 Theoretical background and hypothesis development

### 2.1 Social learning theory

For the foundation of this research, we harness Bandura's social learning theory (SLT) to construct a rigorous theoretical scaffold that connects social media engagement and its allied technologies to academic performance and mental well-being [30].

According to the tenets of SLT, social circles act as crucibles wherein individual competencies and skills predominantly emerge [31]. Further advancing this argument, previous research explicates that external circumstances and peer influences significantly shape learning outcomes [32]. Moreover, this theoretical lens delves into the interplay between intellectual and environmental stimuli in molding both attitudinal and learning dispositions [31]. In this context, learning is conceptualized not as an isolated endeavor, but as a collaborative social activity. In this collaborative era, individuals proactively initiate learning processes, coordinate their intellectual pursuits, and synthesize knowledge through collective cognition [33].

Bandura's seminal theory in 1969 laid the groundwork for understanding how diverse engagements with social networks and collaborative technologies, including social media platforms, materially influence academic performance and learning outcomes [34]. Concretely, AI technologies—through their ability to deliver tailored learning experiences that adapt to individual needs—serve to augment educational engagement and knowledge acquisition [35]. The introduction of Generative AI platforms like ChatGPT into this equation intensifies the need for scrutinizing their latent impact on both academic performance and mental well-being within the student population [3].

Smart learning, as conceptualized through measures such as reading diverse topics, group goal alignment, competitive academic performance, and an affinity for novel technologies and social media applications [36], dovetails with the core tenets of SLT. In smart learning, the affinity for exploring a broad range of subjects reflects the SLT's emphasis on external circumstances and peer influences in shaping learning outcomes [37]. One is not just a passive recipient of information but an active seeker of diverse knowledge. This aligns with Bandura's assertion that individuals in a social setting actively initiate and coordinate their learning processes [38].

Moreover, the measure related to group goal alignment lends itself to SLT's emphasis on collective learning activities. Bandura posited that learning is often a cooperative endeavor within social circles, where mutual goals can significantly influence individual performance and outcomes [32].

The competitive aspect, feeling elated when outperforming peers, can also be linked to SLT. Competition often serves as a motivational mechanism, frequently fueled by peer comparisons, to bolster individual performance—underscoring again the theory's focus on the social environment as a determinant of learning behavior [39].

The inclination to engage with innovative AI technologies like Generative AI (ChatGPT) [40], and new social media applications seamlessly integrates with SLT's scope. Just as SLT acknowledges the role of varied social interactions on learning outcomes, smart learning recognizes the value of technological tools for enhancing academic performance [41]. These tools can serve as an extension of social networks and as customized pedagogical agents that can deeply impact learning outcomes and mental well-being.

To this end, it becomes imperative to dissect how AI can interface with SLT. AI's capacity for customization and interactive content delivery stands as a compelling complement to SLT. Smart learning—through its multifaceted measures—can act as a modern extension of SLT. Through both AI and social media, smart learning can reinforce SLT's social elements while adding the dimension of technological adaptability and a personal drive for excellence. This amalgamation of new-age constructs and SLT offers a robust analytical lens through which we can assess the impacts of AI technologies and social media platforms on academic performance and mental health, mediated by smart learning. Therefore, assessing how AI and social media might bolster academic performance and contribute to mental wellness becomes an important consideration in the broader scholarly dialogue on smart learning and technological interventions in education.

### 2.2 Artificial intelligence and academic performance

AI has emerged as a transformative force in education, significantly bolstering individual learning outcomes [35]. Prior research affirms that AI enhances academic performance through personalized learning experiences, streamlined administrative operations, and targeted support mechanisms [42]. However, to fully realize the benefits of AI in education, we must navigate complex issues related to bias, resource constraints, and ethical considerations [14]. To address these challenges effectively, the multi-disciplinary and multi-stakeholder collaboration among researchers, educators, policymakers, and technology developers is indispensable [43]. Previous work has not only elucidated these challenges but also offered best practices for the responsible integration of AI technologies, while assessing their long-term implications on academic performance and educational success [44].

Generative AI platforms, such as ChatGPT [40], provide students with immersive and experiential learning environments [45]. These technologies simulate real-world conditions, facilitating the practical application of theoretical knowledge and honing students’ problem-solving skills. By immersing students in interactive learning experiences, AI tools like ChatGPT hold the promise to make a meaningful impact on academic performance. Nevertheless, effective deployment of AI in educational settings necessitates comprehensive implementation strategies, faculty training, and rigorous ethical and privacy frameworks [46].

It is imperative to acknowledge that the human element retains its central role in education. AI technologies should act as complements to, not substitutes for, human instruction and mentorship. The integration of Generative AI like ChatGPT into educational systems presents a range of opportunities for enhancing academic performance [4,5]. For example, personalized learning experiences, designed around a student's unique abilities, learning preferences, and interests, can elevate levels of engagement, motivation, and conceptual mastery [42]. Adaptive learning algorithms can diagnose gaps in understanding and deliver targeted interventions, facilitating mastery over key subject areas. Furthermore, AI can liberate educators from the burden of administrative tasks, enabling them to dedicate more time to instructional activities [47].

AI's capacity for generating educational content—such as quizzes, exercises, and study materials—further augments its utility. This automation not only frees up educators' time but also ensures the delivery of high-quality, objective-aligned resources [1]. The transformative potential of AI in academia is not simply about technological sophistication; it is about aligning technology with personalized and adaptive learning experiences to revamp academic performance on a fundamental level.

In striving to deepen our understanding of AI's role in education, we aim to draw connections between its potential advantages, the empowerment of students, improved learning outcomes, and the prospective reconfiguration of educational paradigms. Personalized learning, real-time feedback, intelligent tutoring systems, automated assessments, data-driven analytics, and immersive simulations represent just a few of the mechanisms through which AI can positively shape academic performance [48]. Previous research suggests that sustained focus and effective deployment strategies can further unlock AI's latent potential in bolstering student learning and academic results [45]. Given this discussion, we posit the following hypothesis.

H1 Students believe artificial intelligence has a positive impact on academic performance.

### 2.3 Artificial intelligence and mental well-being

The integration of AI into educational landscapes transcends mere scholastic achievements—it holds the promise of significantly improving students’ mental well-being [49]. Pioneering research [50] has unearthed the myriad ways in which AI can act as a positive force in the mental health landscape, notably in providing emotional support, alleviating stress, facilitating self-reflection, and deploying personalized interventions. AI tools like ChatGPT [3] offer more than informational support; they can serve as non-judgmental platforms for students to articulate their emotional and cognitive struggles [45]. Scholarly inquiries have substantiated the therapeutic potential of AI-based conversational agents, which provide a secure haven for discussing mental and emotional concerns, thereby diminishing stress levels and fostering self-awareness [51].

AI's role is not confined to soft emotional support. A recent study [50] has emphasized AI's utility in healthcare, most compellingly in the early detection and diagnosis of mental health conditions. AI algorithms can scrutinize vast data pools, ranging from electronic health records to social media behavior, for early indicators of mental distress [52]. This proactive approach not only accelerates intervention but also refines treatment strategies, thus enhancing individual outcomes [53]. Virtual agents endowed with AI capabilities can offer a suite of mental well-being services that are both individualized and broadly accessible. These AI-driven interventions range from non-judgmental listening and psychoeducation to evidence-based therapeutic strategies; and together with machine learning algorithms, they can refine these strategies by analyzing treatment-response data, thereby optimizing the likelihood of successful outcomes [54].

AI's promise extends into the realm of immersive technologies. Augmented reality (AR) and virtual reality (VR), when supercharged with AI, can construct rich, immersive environments that have been empirically proven to contribute to relaxation, stress reduction, and a general sense of mental well-being [55]. AI further democratizes educational access for students with special needs. For example, AI-powered transcription services can amplify the academic experience for students with hearing impairments, while content summarization can prove invaluable for those grappling with attention-deficit issues [56]. By annihilating barriers and fostering a more inclusive environment, AI enhances not just academic performance but also contributes materially to students' mental well-being [57].

Nevertheless, the integration of AI into mental well-being interventions requires a nuanced approach. While AI offers unprecedented scalability and personalization, its efficacy should be harmoniously balanced with human expertise to deliver comprehensive mental well-being solutions [58]. An incisive study [59] sheds light on the long-term consequences, ethical dimensions, and best practices for implementing AI in a manner that augments rather than undermines students’ mental well-being. If judiciously deployed, AI possesses the transformative potential to create educational environments that not only facilitate academic excellence but also nurture mental well-being [52]. To encapsulate the above insights into a testable proposition, we articulate the following hypothesis.

H2 Students believe artificial intelligence has a positive impact on mental well-being.

### 2.4 Social media and academic performance

Social media platforms have evolved beyond mere virtual meeting places to become robust ecosystems for information dissemination, learning, and collaboration [60]. Recent research [61] has underscored the potential for social media to catalyze academic activities and collaborations when leveraged responsibly. Another seminal work [62] delved into the enduring impact of social media, examining how shifts in platforms and user behaviors correlate with academic outcomes. However, the utility of social media is not a foregone conclusion; it necessitates prudent time management and purpose-driven utilization to fully harness its educational benefits [63].

Personal dispositions and intrinsic motivations also hold sway in leveraging social media for scholastic advantage. Students equipped with robust self-regulation skills exhibit a higher propensity to harmonize their social media engagement with academic responsibilities, thereby catalyzing their success [46,64]. Research [25] has further nuanced our understanding by pinpointing specific motivators for social media use, such as social connection, entertainment, or information-seeking, each of which carries distinct implications for academic performance.

Contrary to popular apprehension, several studies posit that social media can serve as a facilitator rather than a detractor of academic achievement [65]. Platforms such as Facebook and WhatsApp are not mere distractions but vibrant forums for academic dialogue, facilitating peer-to-peer knowledge exchange, collaborative learning, and enriched comprehension [66]. However, the academic yield from social media is contingent on several variables, including the quality of content, effective time management, and personal discipline in navigating the platform. Excessive or misdirected engagement, particularly with non-academic content, can indeed thwart academic progress. Differentiated usage of social media—whether scrolling through news feeds, engaging in intellectual debates, or joining academically-oriented groups—produces a spectrum of effects on scholastic outcomes [67].

An insightful study [68] further elevates our comprehension of social media's academic potential. The research identifies how social media platforms can act as conduits for students to engage in problem-solving and interact with peers for scholastic pursuits. As a result, students are not just passive consumers but active participants in their educational journey. In synthesizing these findings, the study culminates in the formulation of the ensuing hypothesis.

H3 Students believe social media has a positive impact on academic performance.

### 2.5 Social media and mental well-being

Mental well-being encompasses a harmonious blend of psychological efficacy and positive emotional state [69]. The omnipresence of social media in contemporary society furnishes individuals with an expansive toolkit for social engagement, information dissemination, and personal advocacy [70]. Recent research [11] underscores that students experience heightened mental well-being when they deploy social media for educational advancement. This positive trajectory manifests through elevated psychological acuity, fortified by familial support and smart learning techniques [71]. Notably, the confluence of psychological well-being and goal-oriented environmental mastery significantly augments academic outcomes [72].

Social media also serves as an invaluable scaffold for university students, offering avenues to forge social networks and foster dialogue that ameliorates mental strain [73]. The existential gratification stemming from academic achievements further amplifies this sentiment [74]. Social media's repository of resources functions as a catalytic agent for academic progression [75], and remaining current with news and topical discussions enhances students' overall well-being [76]. An illuminating study [77] reveals that social media organizations and online communities engender a sense of belonging among young individuals, structured around common academic or recreational interests.

Yet, the influence of social media on mental well-being is not unidirectional nor unequivocally positive [78]. A critical examination [79] ascertains the manifold benefits of social media, such as collaborative learning and resource accessibility. Prior work [80] has also validated the positive implications of recreational social media use for mental well-being, while other work [81] zeroes in on the roles of usage frequency and quality. However, the empirical landscape presents a mosaic of findings concerning the impact of social media on indicators of mental health, such as anxiety, depression, loneliness, and low self-esteem [82]. Nonetheless, it is noteworthy that social media platforms offer not merely distractions but reservoirs of mental well-being resources, such as stress management techniques and professional consultation avenues [83]. This cornucopia of resources emboldens students to seize control over their mental well-being proactively. Taken collectively, these disparate outcomes call for a nuanced evaluation of the intricate relationship between social media use and mental well-being. Thus, in light of these multiple dimensions, our study advances the following hypothesis.

H4 Students believe social media has a positive impact on mental well-being.

### 2.6 Mediating role of smart learning

Smart learning epitomizes the convergence of digital technologies—including AI and social media—in reconfiguring educational landscapes into more dynamic and efficient learning ecosystems [84]. This pedagogical evolution manifests through customized, interactive learning experiences, fortified by the analytical prowess of AI and the social utility of media platforms [85]. Smart learning environments empower students with unparalleled access to educational resources, collaborative platforms, and personalized feedback loops, thereby enriching self-directed learning [86].

Educational institutions are leveraging smart learning to boost student engagement, foster collaborative ecosystems, promote mental well-being, and strengthen academic performance [87]. Universities, in particular, are seizing the technological dividend of smart learning to cultivate engaging and inclusive educational sanctuaries that amplify both student potential and well-being [26]. A seminal study [88] found that smart learning interventions exert a positive impact on students’ academic performance and mental well-being. Both AI and social media can serve as catalysts for creating vibrant, smart learning ecosystems that elevate student motivation, self-efficacy, and sense of community [89]—factors paramount for academic achievement and mental well-being.

Technologies such as Generative AI and social media platforms can synergistically amplify the impact of smart learning by facilitating communal knowledge transfer, online discourse, and resource-sharing [90]. ChatGPT, a constituent of the AI ecosystem, can act as an enabler for active learning by promoting real-time engagement and fluid knowledge exchange, thereby influencing academic outcomes [91]. Specifically, AI's data-driven personalization can augment the educational experience by offering intelligent feedback and adaptive course material [92], wherein such personalized scaffolding holds immense potential for enhancing student comprehension, engagement, and overall academic performance.

Social networking platforms, integrated into smart learning environments, can offer emotional support and intellectual enrichment by fostering peer relationships and disseminating mental well-being insights [93]. Smart learning can incite intellectual curiosity and problem-solving agility in students by harnessing the innovative potential of AI [94]. Individuals engage in smart learning for pioneering ventures, thereby enriching educational contexts with novel scholarly contributions [95]. When integrated, these platforms produce a synergistic effect that enhances students' educational experiences and mental well-being [96]. Notably, research [97] underscores the pivotal role of AI-driven smart learning in augmenting students’ academic performance. In light of these intricate dynamics, our study advances the following hypotheses.

H5 Smart learning positively mediates the relationships of student perceptions between AI with academic performance (**H**_**5a**_) and mental well-being (**H**_**5b**_).

H6 Smart learning positively mediates the relationships of student perceptions between social media with academic performance (**H**_**6a**_) and mental well-being (**H**_**6b**_).

**Fig. 1:**
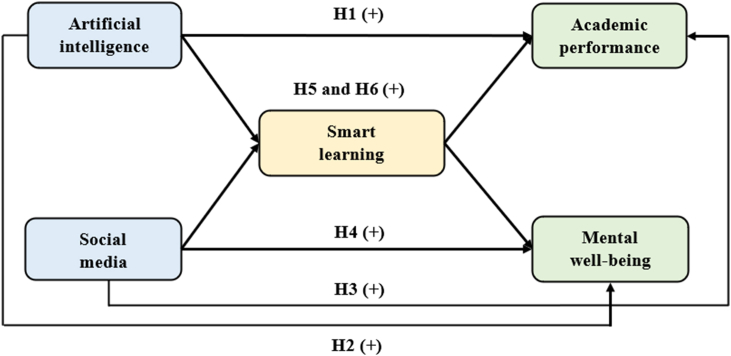
Conceptual framework.

Fig. 1 delineates the interplay among these variables, showcasing AI and social media as independent variables (antecedents), smart learning as the mediating variable (facilitator), and academic performance and mental well-being as the dependent variables (consequences).

## 3 Methodology

### 3.1 Approach

AI and social media have revolutionized communication and knowledge exchange, transforming the ways individuals engage with information and each other [98]. These platforms offer rich environments for smart learning, enabling seamless knowledge exchange between students and educators beyond traditional educational settings [99]. Given the pervasiveness of technology among today's youth, who frequently leverage AI and social media for academic pursuits, this study adopts a realist research philosophy with a deductive approach to investigate how university students look at the impact of these technological platforms on academic performance and mental well-being [100]. Notably, deductive reasoning allows us to test hypotheses derived from established theories, frequently using quantitative methods that align well with hypothesis development [101].

### 3.2 Instrumentation

In this study, we employed a strategic distribution of questionnaires to collect data. The constructs or variables under examination were gauged using scales refined from prior research, tailored specifically to suit our sample [102]. We adopted the Likert scale to elicit nuanced responses, enabling us to discern the intensity of the respondents’ agreement or disagreement with given statements. Specifically, a five-point Likert scale was selected to capture a broader spectrum of attitudes, offering respondents a greater range of options [103].

#### 3.2.1 Artificial intelligence

AI encompasses the design of algorithms and computational systems that mimic human-like cognitive functions such as reasoning, problem-solving, and pattern recognition [104]. We employed an eight-item scale for AI, borrowed from seminal works in the field [105,106]. Consistent with our methodological approach, a five-point Likert scale was utilized to gauge all AI-related items.

#### 3.2.2 Social media

Social media, defined as online platforms for creating, disseminating, and interacting with user-generated content within virtual communities [107], play a pivotal role in our research. We adapted a six-item scale from an existing study that focused on students [16]. To quantify the responses, we deployed a five-point Likert scale, ranging from 1 (strongly disagree) to 5 (strongly agree) for each social media item under investigation.

#### 3.2.3 Smart learning

Smart learning is an educational paradigm that leverages cutting-edge technologies, including AI and social media, to enrich students' learning experiences and outcomes [108]. We incorporated a five-item scale sourced from prior research [90]. In alignment with the overall methodology, a five-point Likert scale, ranging from 1 (strongly disagree) to 5 (strongly agree) was used to evaluate the respondents’ perspectives on all smart learning items.

#### 3.2.4 Academic performance

Academic performance denotes the student's scholastic achievements and is a cornerstone of educational research [60]. We used an established scale developed by authoritative researchers [10] to assess students' academic performance. A five-point Likert scale served as the measuring stick, offering options from 1 (strongly disagree) to 5 (strongly agree) for all academic performance items.

#### 3.2.5 Mental well-being

Contrary to the reductive view that equates mental well-being merely with the absence of mental illness, we adopt a more holistic lens that encompasses emotional positivity, purposefulness, resilience, and life satisfaction [72]. We selected a six-item scale for mental well-being from an earlier study [57]. To gauge these dimensions, we utilized a five-point Likert scale, ranging from 1 (strongly disagree) to 5 (strongly agree), thus maintaining consistency in our methodological design.

### 3.3 Sampling

For data collection, we randomly selected six top-ranking universities in Beijing, China, as listed by Times Higher Education. The choice of university students as the unit of analysis is strategic, given their active engagement with the technologies under scrutiny [109,110]. Owing to the indeterminate size of the student population, a convenience sampling technique was employed to select the respondents [103]. The data was gathered using a structured survey administered to university students. We ensured robust participation by disseminating the survey's questionnaire both in random classes on university campuses and through online platforms (wenjuanxing-sojump). A total of 495 questionnaires were distributed and 427 completed questionnaires were returned. This data collection phase unfolded between March and May 2023 using a cross-sectional design. All questionnaires returned underwent rigorous screening for missing values, multivariate outliers, and unengaged responses. Subsequently, 26 cases were discarded, leaving 401 useable cases for analysis—equating to an 81 % useable response rate. This sample size aligns with guidelines suggesting that empirical research should involve more than 30 but fewer than 500 participants [111].

The demographic profile of the respondents provides a nuanced view of the study's target population. Males constituted 54 %, while females accounted for 46 % of the sample. With regard to age, 43 % fell within the 18 to 21 age brackets, 52 % in the 22 to 28 categories, and 5 % in the 29 to 35 range. Educational background varied: 22 % held diplomas, 30 % had bachelor's degrees, 42 % had master's degrees, and 5 % possessed doctoral degrees. Regarding daily internet use, 18 % reported using the internet for 1–4 h, 48 % for 4–8 h, and 34 % for 8–12 h specifically for academic and problem-solving purposes. The demographic particulars are comprehensively detailed in Table 1.

**Table 1:** Profile of respondents.

Demographic	Frequency (*n*: 401)	Percentage (%: 100)
**Gender**		
Female	184	46 %
Male	217	54 %
**Age**		
18–21 years	171	43 %
22–28 years	211	52 %
29–35 years	19	5 %
More than 35 years	0	0 %
**Education**		
Diploma	89	22 %
Bachelor degree	121	30 %
Master degree	170	42 %
Doctoral degree	21	5 %
**Daily internet use for academic and problem-solving purposes**		
1–4 h	71	18 %
4–8 h	191	48 %
More than 8 h	139	34 %

### 3.4 Analysis

Partial least square-structural equation modeling (PLS-SEM) serves as an analytical technique for probing intricate relationships between variables within a given theoretical framework [112]. This robust, second-generation regression analysis synergizes confirmatory factor analysis (CFA) and multiple linear regression, thereby permitting the concurrent employment of both measurement and structural models [113]. To rigorously assess these models, this study deployed the specialized statistical software SmartPLS. The selection of PLS-SEM as the analytical technique of choice in this investigation was not arbitrary; it was predicated on its pervasive application and demonstrated utility in extant literature [114,115]. Compared to conventional statistical methods, SEM delivers superior outcomes by enhancing the efficiency and rigor of statistical analysis [116].

## 4 Results

### 4.1 Common method bias

To rigorously evaluate the potential for common method bias in this study, we employed Harman's one-factor test utilizing SPSS software [115]. This approach continues to be recommended by contemporary research for its simplicity and diagnostic utility, notwithstanding its acknowledged limitations [117]. Our analysis revealed that a single component accounted for a mere 19 % of the variance, falling substantially below the conventional 50 % threshold indicative of common method bias [118]. These findings strongly suggest that common method bias does not compromise the integrity of this investigation.

### 4.2 Measurement model

The measurement model serves as a rigorous framework to substantiate the dependability of the constructs and to authenticate their validity [119,120]. Utilizing PLS-SEM, this study meticulously assessed key metrics such as Cronbach's alpha (α), composite reliability (CR), and average variance extracted (AVE) [116] alongside the Fornell-Larcker criteria [121], the heterotrait-monotrait (HTMT) ratio [122], and the variance inflation factor (VIF) [102].

The pursuit of internal consistency was executed through Cronbach's α and CR. These metrics serve as the linchpins of construct reliability, illuminating the degree of correlation among items within each construct. A threshold value 0.7 has been established as the benchmark for construct reliability [123]. As evidenced by the data, each variable surpassed this threshold: Cronbach's α values were 0.960 for artificial intelligence, 0.871 for social media, 0.924 for smart learning, 0.975 for academic performance, and 0.971 for mental well-being. Similarly, CR scores were 0.966 for artificial intelligence, 0.903 for social media, 0.940 for smart learning, 0.980 for academic performance, and 0.978 for mental well-being. As delineated in Table 2, each construct boasted a Cronbach's α and CR score exceeding 0.7, exemplifying robust internal consistency.

**Table 2:** Measurement model results.

Construct	Items		FL	α	CR	AVE	Source
Artificial intelligence (AI)				0.960	0.966	0.779	[105,106]
	AI1	AI-based systems like ChatGPT improve learning performance.	0.846				
	AI2	AI-based systems like ChatGPT improve learning efficiency.	0.869				
	AI3	AI-based systems like ChatGPT improve learning outcomes.	0.787				
	AI4	AI-based systems like ChatGPT improve collaborative endeavors.	0.922				
	AI5	AI-based systems like ChatGPT optimize the learning process.	0.908				
	AI6	AI-based systems like ChatGPT are impressive with their multifaceted capabilities.	0.912				
	AI7	AI-based systems like ChatGPT are invaluable for enriching the learning experience.	0.898				
	AI8	AI-based systems like ChatGPT improve the precision of inquiries when they are used to ask targeted follow-up questions.	0.910				
Social media (SM)				0.871	0.903	0.609	[16]
	SM1	Social media platforms elevate the sense of community and interactivity in learning.	0.753				
	SM2	Social media platforms enable faster peer feedback.	0.847				
	SM3	Social media platforms accelerate instructor feedback.	0.845				
	SM4	Social media platforms amplify class participation.	0.773				
	SM5	Social media platforms facilitate effective multitasking during study sessions.	0.722				
	SM6	Social media platforms serve as robust channels for effective academic communication.	0.732				
Smart learning (SL)				0.924	0.940	0.758	[90]
	SL1	With smart technologies, I find myself learning diverse topics.	0.867				
	SL2	With smart technologies, I find myself engaging in group learning.	0.900				
	SL3	With smart technologies, I find myself performing better than others.	0.899				
	SL4	With smart technologies, I find myself enjoying learning about new topics.	0.823				
	SL5	With smart technologies, I find myself enjoying learning about new applications.	0.860				
Academic performance (AP)				0.975	0.980	0.908	[10]
	AP1	I am making progress in my quantity of study.	0.926				
	AP2	I am making progress in the quality of my studies.	0.935				
	AP3	I am making progress in my personal career goals.	0.962				
	AP4	I am making progress in developing the skills needed for my future career.	0.978				
	AP5	I am making progress in seeking out career development opportunities.	0.963				
Mental well-being (MWB)				0.971	0.978	0.880	[57]
	MWB1	I have been feeling optimistic.	0.946				
	MWB2	I have been feeling relaxed.	0.974				
	MWB3	I have been dealing with problems well.	0.981				
	MWB4	I have been thinking clearly.	0.969				
	MWB5	I have been feeling close to other people.	0.972				
	MWB6	I have been able to make up my mind about things.	0.767				

**Notes:** FL = Factor loading. α = Cronbach's alpha. CR = Composite reliability. AVE = Average variance extracted.

Convergent validity was another metric scrutinized, gauged by the outer loading of each construct as well as by the average variance extracted (AVE). The AVE values for artificial intelligence, social media, smart learning, academic performance, and mental well-being were 0.779, 0.609, 0.758, 0.908, and 0.880, respectively. These findings not only meet but exceed the 0.5 threshold for AVE, thereby confirming convergent validity. Factor loadings further elucidate the proportion of variance attributable to each variable within a specific factor. Adhering to the standards of SEM, factor loadings above 0.7 are strongly encouraged [103]. Table 2 corroborates that all variables met this criterion, further affirming the model's convergent validity.

Discriminant validity was ascertained using the Fornell and Larcker criteria, which ensures that each variable is distinctly separate from all others within the same construct [121]. A stringent threshold requires that the square root of the AVE must surpass the correlation values among competing variables. As Table 3 attests, the discriminant validity of each variable exceeded 0.7, thereby fulfilling the stipulated criteria [123]. Additionally, this study employed the HTMT ratio to evaluate the similarity among latent constructs. With a standard HTMT range between −1 and 1, the research confirmed discriminant validity for all variables, as indicated in Table 4. These HTMT ratios were all less than 0.85, adhering to best practices for discriminant validity [122].

**Table 3:** Discriminant validity assessment through Fornell-Larcker.

Construct	AI	SM	SL	AP	MWB
Artificial intelligence (AI)	**0.883**				
Social media (SM)	0.038	**0.780**			
Smart learning (SL)	0.060	0.572	**0.870**		
Academic performance (AP)	0.085	0.536	0.409	**0.953**	
Mental well-being (MWB)	0.087	0.538	0.412	0.958	**0.938**

**Table 4:** Discriminant validity assessment through Heterotrait-Monotrait (HTMT) ratio.

Construct	AI	SM	SL	AP	MWB
Artificial intelligence (AI)					
Social media (SM)	0.082				
Smart learning (SL)	0.059	0.600			
Academic performance (AP)	0.084	0.571	0.403		
Mental well-being (MWB)	0.084	0.576	0.403	0.830	

Finally, the VIF served as a diagnostic tool to examine the degree of multicollinearity through regression analysis. All variables in this study met the threshold criteria of VIF <5, further fortifying the rigor of the measurement model [102].

**Fig. 2:**
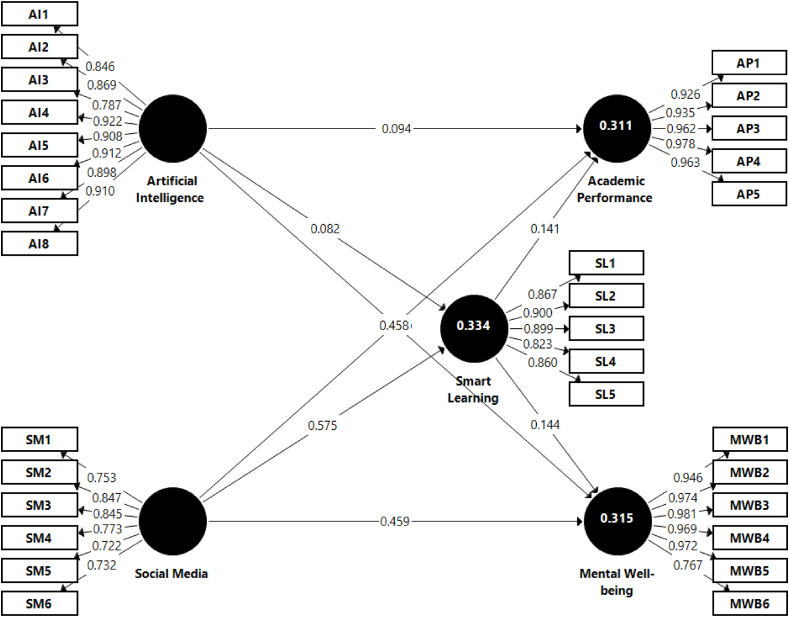
Structural model.

Taken collectively, the measurement model passed rigorous tests for reliability and validity, positioning it as a robust measure for evaluating the relationships among the constructs or variables under investigation. The detailed factor loadings and measurement model are graphically elucidated in Fig. 2.

### 4.3 Structural model

After conducting a rigorous evaluation of the reliability and validity tests through the measurement model, we proceed to the structural model with PLS-SEM. This model serves as the analytical crucible where the relationships among variables are systematically examined for hypothesis testing [102]. A plethora of statistical metrics, including the effect size (*f*^2^), *t*-values, predictive relevance (*Q*^2^), coefficient of determination (*R*^2^), and path coefficients, are rendered salient by this structural model [123].

Our hypotheses articulate those students believe AI and social media exert a positive influence on both academic performance and mental well-being. Furthermore, this influence is mediated by the role of smart learning. Utilizing bootstrapping within the SmartPLS software, we gauge the statistical significance of these relationships. This methodological step illuminates not only the directionality (positive or negative) but also the magnitude and significance of the influence exerted by independent variables on dependent variables [124].

Our evidence robustly supports H1, indicating that students perceive a positive relationship between student perceptions of AI and academic performance (*β* = 0.094, *t* = 2.335, *p* < 0.05). Similarly, H2 is validated, showing that students also perceive AI as positively influencing mental well-being (*β* = 0.096, *t* = 2.299, *p* < 0.05). Substantiating [97], we find that students perceive social media, too, has a positive impact on academic performance (*β* = 0.458, *t* = 9.321, *p* < 0.001). H4 further buttresses this narrative by demonstrating a positive relationship between social media and mental well-being (*β* = 0.459, *t* = 9.654, *p* < 0.001). Notably, students perceive both AI (*β* = 0.082, *t* = 2.092, *p* < 0.05) and social media (*β* = 0.575, *t* = 17.271, *p* < 0.001) also exert a positive influence on smart learning. Students believe smart learning, in turn, positively affects both academic performance (*β* = 0.141, *t* = 2.781, *p* < 0.01) and mental well-being (*β* = 0.144, *t* = 2.926, *p* < 0.01). Thus, H1 through H4 are unequivocally supported, as detailed in Table 5.

**Table 5:** Structural model.

Relationship	β-value	Standard deviation	*t*-value	*p*-value	Outcome
**Direct effect**					
Artificial intelligence → Academic performance (H1)	0.094	0.041	2.335	0.020	Supported
Artificial intelligence → Mental well-being (H2)	0.096	0.042	2.299	0.022	Supported
Social media → Academic performance ([97])	0.458	0.046	9.321	0.000	Supported
Social media → Mental well-being (H4)	0.459	0.046	9.654	0.000	Supported
Artificial intelligence → Smart learning	0.082	0.040	2.092	0.037	
Social media → Smart learning	0.575	0.034	17.271	0.000	
Smart learning → Academic performance	0.141	0.050	2.781	0.007	
Smart learning → Mental well-being	0.144	0.048	2.926	0.004	
**Mediation effect**					
Artificial intelligence → Smart learning → Academic performance (H5a)	0.092	0.028	3.285	0.022	Supported
Artificial intelligence → Smart learning → Mental well-being (H5b)	0.132	0.048	2.754	0.030	Supported
Social media → Smart learning → Academic performance (H6a)	0.082	0.030	2.712	0.007	Supported
Social media → Smart learning → Mental well-being (H6b)	0.084	0.028	2.961	0.003	Supported

In the case of H5, our analysis reveals that smart learning serves as a positive mediator between AI and both academic performance (*β* = 0.092, *t* = 3.285, *p* < 0.05) and mental well-being (*β* = 0.132, *t* = 2.754, *p* < 0.05). Similarly, H6 follows suit, indicating that smart learning also positively mediates the relationship between social media and both academic performance (*β* = 0.082, *t* = 2.712, *p* < 0.01) and mental well-being (*β* = 0.084, *t* = 2.961, *p* < 0.01). Thus, both H5 and H6 are supported.

However, it is prudent to note that relying solely on the *R*^2^ value as an evaluation metric presents limitations [125]. In our model, the *R*^2^ values stand at 0.334 for smart learning, 0.311 for academic performance, and 0.315 for mental well-being. Consequently, the analytical spotlight shifts to the *Q*^2^ metric, which holds greater prognostic utility and validity [126]. A *Q*^2^ value exceeding zero signifies high predictive relevance. Our model, with a *Q*^2^ of 0.495, showcases students' belief in its robust predictive power for improving both academic performance and mental well-being. As per guidelines [127], typical *f*^2^ values of 0.03, 0.19, and 0.491 denote small, medium, and large effects, respectively. Our analysis reveals that the effect sizes in our study range from moderate to large [126].

The comprehensive array of these statistical techniques and outcomes is cataloged in Table 5, and the structural model is visually represented in Fig. 2.

## 5 Discussion

AI and social media have woven themselves deeply into the fabric of modern life, redefining paradigms in sectors ranging from education to interpersonal communication [128]. These advancements have brought immeasurable benefits but have also raised nuanced questions about their impacts on academic performance and mental well-being [51]. Recognizing the gravity of these concerns, this study aimed to rigorously explore students' perceptions of the interplay among AI, social media, academic performance, and mental well-being within the context of smart learning. Through empirical analysis of 401 university students, the investigation culminated in six pivotal conclusions.

Firstly, our research confirms that students believe AI exerts a positive influence on academic performance. AI's transformative capacity has garnered considerable scholarly attention, particularly in the realm of education [23]. Our work resonates with prior research [48], demonstrating AI's capacity to enhance learning experiences, enable curriculum personalization, and improve educational outcomes. AI technologies, including machine learning, offer the education sector unparalleled capabilities to analyze vast datasets, thereby providing actionable insights to optimize student performance [12,45]. This aligns with extant literature affirming the benefits of emergent technologies in education [4,20,129].

Secondly, our study reveals that students believe AI can also positively affect mental well-being. Although previous studies have flagged potential risks [130,131], our research underscores AI's promise in enhancing mental health support systems [132]. Through AI-driven chatbots and virtual assistants, immediate support and vital information become more accessible, thereby democratizing mental health services [1]. We argue that for AI to continue to be a force for good, ethical frameworks and human oversight are non-negotiable [57]. These findings are substantiated by prior research on AI's impact on mental well-being [56,101].

Thirdly, our findings illuminate those students believe social media can be a net positive for academic performance. While social media's intrusive nature has raised concerns [30], our study affirms its utility as a tool for knowledge-sharing and collaborative learning [133,134]. Additionally, social media serves as a conduit for networking with professionals, experts, and mentors, enriching both personal and academic development [30]. Our study harmonizes with existing scholarship, suggesting that the key to unlocking social media's academic potential lies in self-discipline and time management [60,135].

Fourthly, the results suggest that students believe social media also enhances mental well-being. Active engagement on these platforms correlates with heightened emotional support and social connectedness [136], thus mitigating feelings of isolation and loneliness. Previous studies corroborate the transformative power of social media in fostering mental resilience among marginalized communities [137]. These positive interactions amplify life satisfaction, self-esteem, and overall happiness, as indicated in existing literature [8,[138], [139], [140]].

Fifthly, our study elucidates that smart learning plays a mediating role in students' beliefs between AI and both academic performance and mental well-being. Smart learning involves personalized, adaptive learning experiences [141]. The incorporation of AI technologies, such as intelligent tutoring systems and virtual simulations, are symptomatic of a burgeoning educational landscape optimized for individual student needs [104,142]. These findings gain further credibility from extant literature [24,93].

Lastly, we find that smart learning also serves as a mediator in students' beliefs between social media and the twin outcomes of academic performance and mental well-being. The widespread adoption of social media among students enriches both their scholastic and emotional lives [143]. Factors like effective learning strategies, high levels of engagement, and abundant educational resources contribute to this positive trend [95]. This aligns seamlessly with prior studies highlighting the efficacy of smart learning in concert with social media usage [85,88].

In culmination, our research not only affirms but also builds upon the extant literature, offering a nuanced understanding of the intricate relationships among AI, social media, academic performance, and mental well-being in students' belief matrices in the age of smart learning. This scholarly contribution lays a foundation for future research aimed at maximizing the societal benefits of these transformative technologies.

## 6 Conclusion

This study examined student perceptions of the impact of artificial intelligence and social media on academic performance and mental well-being through smart learning in Chinese university students. The results revealed six significant findings. First, students believe artificial intelligence has a significant effect on the academic performance of Chinese students. Second, students believe artificial intelligence significantly affects students' mental well-being. Third, students believe social media positively impacts the academic performance. Fourth, students believe social media positively impacts their mental well-being. Fifth, smart learning positively mediates the relationship between artificial intelligence, academic performance, and mental well-being. Six, smart learning positively mediates the relationship between social media, academic performance, and mental well-being. In conclusion, this study offers a compelling empirical foundation for policy formulation in the intricate nexus of AI, social media, academic performance, and mental well-being in the era of smart learning. Policymakers are provided with a comprehensive, evidence-based roadmap to navigate the complex landscape of technological innovation and its societal implications. This policy guidance not only complements our academic contribution but also aims to catalyze actionable change in the pursuit of human-centric technology adoption.

### 6.1 Theoretical and practical implications

The discussion of theoretical and practical implications serves as a vital extension of our academic findings, transitioning from scholarly insight to actionable guidance.

In the context of higher education, our research underlines the urgent need for institutions to integrate AI into their educational models. Universities should not just view AI as an add-on but as a transformative tool that can personalize education, automate routine administrative tasks, and offer robust data analytics for academic improvement. By incorporating AI-driven systems, educational institutions can foster a learning environment that is both dynamic and responsive to individual student needs and preferences. This could ultimately serve the dual purpose of boosting academic performance while also contributing positively to students’ mental well-being.

The demonstrated benefits of AI in mental health provision suggest an immediate avenue for its ethical deployment in healthcare services. Medical professionals and counsellors at educational institutions could collaborate with technologists to develop AI-driven mental health platforms. These platforms could offer immediate, stigmatization-free initial contact points for individuals who may be reluctant to seek traditional forms of mental health support. To that end, healthcare providers and policy makers ought to consider investing in AI-driven technologies that deliver instant, quality mental health services at scale.

Turning our attention to social media, and its perceived positive impact on academic performance and mental well-being suggests that rather than instituting blanket bans or restrictions, educational institutions should consider how to integrate social media effectively into educational pedagogies. For example, educators could use social media platforms to disseminate course materials, encourage group discussions, and foster academic collaboration. This approach requires a change in institutional mindset to see social media not as a distraction but as a modern tool for learning and interaction. Also, the power of social media to facilitate professional networking should not be overlooked; career services should embrace this potential and educate students on how to cultivate professional relationships online.

The mediating role of smart learning in both AI and social media applications also calls for a new blueprint in educational strategy. The implication here is that smart learning could serve as a unifying mechanism that combines the advantages of AI and social media. Education administrators should consider deploying smart learning systems as an integrated part of the education process, one that can collate various data points to provide a more rounded, holistic educational experience.

In conclusion, the practical implications of our study are both broad and profound, touching on sectors ranging from education and healthcare to technological development and public policy. Our findings offer a roadmap for stakeholders in these sectors to make informed, data-driven decisions that capitalize on the benefits of AI and social media while also being cognizant of their potential pitfalls. The overarching aim should be to enhance both the academic and mental well-being of students, thereby enriching the quality of education and life in the modern age.

### 6.2 Policy implications

The policy implications of this research are both salient and far-reaching. As AI and social media technologies continue to evolve at an unprecedented pace, their integration into educational and mental well-being sectors presents a pressing need for evidence-based policy guidance. Our findings, grounded in rigorous empirical analysis, offer a nuanced framework for policymakers to construct policies that strike a delicate balance between technological innovation and human welfare.

One of the most immediate areas for policy intervention is the incorporation of AI in educational systems. Our research confirms that students believe AI has the potential to significantly elevate academic performance. In this regard, policymakers should consider not only the allocation of resources for AI integration but also the development of national standards for AI in educational contexts. Such standards could set forth guidelines for curriculum personalization, data ethics, and student privacy, all while ensuring that AI serves as a tool for educational equity rather than a catalyst for division. Existing frameworks for educational standards can be adapted to include AI-specific guidelines, informed by this and other scholarly work [23,144].

Turning our attention to student perceptions of mental well-being, and student preferences for AI warrant policy actions aimed at expanding accessibility to AI-powered mental health resources. Policymakers should explore partnerships with technology companies and healthcare providers to democratize access to AI-driven mental health tools, such as chatbots and virtual therapists. Regulations must be put in place to ensure the ethical use of data and to provide guidelines for human oversight. Given that AI in healthcare is an emerging field, agile policy frameworks are essential to adapt to the rapid advancements in technology, without sacrificing rigorous standards for ethics and efficacy [57,131].

Similarly, student perceptions of the social media in academic and mental well-being contexts also invite focused policy scrutiny. Although our study found positive impacts, it is imperative for policymakers to also consider potential negative externalities such as cyberbullying, privacy breaches, and misinformation [40,145]. Educational institutions could benefit from policy guidelines on the responsible use of social media within academic settings [3]. Such guidelines could encourage constructive online discourse and information sharing while curbing distractions and other detriments to academic focus [133,134].

Lastly, the mediating role of smart learning in enhancing student perceptions of both academic performance and mental well-being necessitates a policy that supports its integration in educational and mental health contexts. Funding allocations for research and development in smart learning technologies, as well as the formulation of best practice guidelines, can be particularly useful. The policy could advocate for a multi-stakeholder approach involving educators, technologists, and mental health professionals, thereby ensuring a holistic strategy.

### 6.3 Limitations and future directions

This investigation, while comprehensive, is not without its limitations, each of which suggests avenues for future research that could enhance the robustness and generalizability of our findings.

To begin, the cultural and geographic scope of this study is limited to young university students, primarily from Beijing, China. This specificity renders our conclusions potentially less applicable to young individuals in other cultural or demographic contexts. Future studies should expand the participant pool to include students from varied cultural backgrounds and educational systems, thereby enriching the cross-cultural validity of the results.

Additionally, the study's sample characteristics also limit its generalizability. Our findings draw upon data from a relatively homogeneous group of participants—namely, young students attending universities in Beijing, China. As such, the outcomes may not be representative of the broader young population in China, let alone in other countries. Subsequent research endeavors should aim for a more diverse sampling strategy that considers various socio-economic statuses, educational environments, and perhaps even age groups, within both China and other countries.

Besides that, the methodology employed in this study is based on self-reporting techniques involving surveys rather than actual student tests, grades, or assessments, which are susceptible to a variety of biases, such as social desirability and memory recall issues. These biases could potentially skew the data, diluting the rigor of our conclusions. Future research could mitigate this limitation by adopting a longitudinal research design, enabling more accurate and temporally sensitive data collection. Alternatively, combining self-report methods with objective metrics might offer a more holistic view.

Furthermore, while our study emphasizes the mediating role of smart learning in relation to academic performance, there are other potential mediators that remain unexplored. Future research could consider integrating variables like student engagement and motivation as mediating factors. These additional layers of analysis could offer a more nuanced understanding of how students perceive smart learning interfaces with academic performance and mental well-being.

Moreover, the technology utilized in our study was primarily Generative AI—specifically, ChatGPT—to enhance academic performance and mental well-being. While this approach offers significant insights, it also narrows the technological landscape under examination. Future work could benefit from incorporating a broader array of AI technologies, perhaps even including machine learning algorithms or predictive analytics, to gauge their relative efficacy in improving student learning outcomes.

Last but not least, a noteworthy limitation of our study is the absence of control groups, which makes it challenging to draw definitive causal inferences regarding the impact of AI and social media on students’ academic performance and mental well-being. Future research would benefit substantially from including control groups, thereby strengthening the causal inferences that can be drawn from the study results. This methodological refinement would serve to bolster the empirical rigor of subsequent investigations.

To this end, by acknowledging these limitations and outlining future research directions, we endeavor to contribute to the ongoing scholarly dialogue in a manner that is both transparent and constructive. Further studies in these directions will not only fortify the scientific understanding of the interconnected domains explored here but will also inform educational policies and mental health interventions for the betterment of society at large and the future of education and work [3].

## Funding

This work received financial support from the 10.13039/501100001809National Natural Science Foundation of China under grant number 72074014.

## Data availability statement

Data will be made available on request.

## CRediT authorship contribution statement

**Muhammad Farrukh Shahzad:** Writing – review & editing, Writing – original draft, Conceptualization, Formal analysis, Methodology, Project administration, Software, Validation, Visualization. **Shuo Xu:** Writing – review & editing, Writing – original draft, Supervision, Resources, Investigation, Funding acquisition, Conceptualization. **Weng Marc Lim:** Resources, Project administration, Investigation, Writing – original draft, Writing – review & editing. **Xingbing Yang:** Validation, Software. **Qasim Raza Khan:** Validation, Supervision, Methodology.

## Declaration of competing interest

The authors declare the following financial interests/personal relationships which may be considered as potential competing interests: Shuo Xu reports financial support was provided by National Natural Science Foundation of China. If there are other authors, they declare that they have no known competing financial interests or personal relationships that could have appeared to influence the work reported in this paper.

## References

[1] Hu B., Mao Y., Kim K.J. (2023). How social anxiety leads to problematic use of conversational AI: the roles of loneliness, rumination, and mind perception. Comput. Human Behav..

[2] Edward H. (2023). Artificial intelligence and obesity management : an Obesity Medicine Association (OMA) clinical practice statement (CPS) 2023. Obes. Pillars.

[3] Ray P.P. (2023). ChatGPT: a comprehensive review on background, applications, key challenges, bias, ethics, limitations and future scope. Internet Things Cyber-Physical Syst.

[4] Yu H., Guo Y. (2023). Generative artificial intelligence empowers educational reform: current status, issues, and prospects. Front. Educ..

[5] Dimitriadou E., Lanitis A. (2023). A critical evaluation, challenges, and future perspectives of using artificial intelligence and emerging technologies in smart classrooms. Smart Learn. Environ.

[6] Koyuturk C. (2023). http://arxiv.org/abs/2306.10645.

[7] Ouyang F., Wu M., Zheng L., Zhang L., Jiao P. (2023). Integration of artificial intelligence performance prediction and learning analytics to improve student learning in online engineering course. Int. J. Educ. Technol. High. Educ..

[8] Tlili A. (2023). What if the devil is my guardian angel: ChatGPT as a case study of using chatbots in education. Smart Learn. Environ.

[9] Sanchez Reina J.R., Theophilou E., Ognibene D., Davinia H.-L. (December, 2023).

[10] Whelan E., Islam A.K.M.N., Brooks S. (2020). Applying the SOBC paradigm to explain how social media overload affects academic performance. Comput. Educ..

[11] Whelan E., Golden W., Tarafdar M. (2022). How technostress and self-control of social networking sites affect academic achievement and wellbeing. Internet Res..

[12] André Q. (2018). Consumer choice and autonomy in the age of artificial intelligence and big data. Cust. Needs Solut..

[13] Chassignol M., Khoroshavin A., Klimova A., Bilyatdinova A. (2018). Artificial Intelligence trends in education: a narrative overview. Procedia Comput. Sci..

[14] Alqahtani T. (May, 2023). The emergent role of artificial intelligence, natural learning processing, and large language models in higher education and research. Res. Soc. Adm. Pharm..

[15] Alwagait E., Shahzad B., Alim S. (2015). Impact of social media usage on students academic performance in Saudi Arabia. Comput. Human Behav..

[16] Lau W.W.F. (2017). Effects of social media usage and social media multitasking on the academic performance of university students. Comput. Human Behav..

[17] Giunchiglia F., Zeni M., Gobbi E., Bignotti E., Bison I. (May 2018). Mobile social media usage and academic performance. Comput. Human Behav..

[18] Craig W. (2020). Social media use and cyber-bullying: a cross-national analysis of young people in 42 countries. J. Adolesc. Heal..

[19] Delanerolle G. (2021). Artificial intelligence: a rapid case for advancement in the personalization of Gynaecology/Obstetric and Mental Health care. Women’s Heal..

[20] Brougham D., Haar J. (2018). Smart technology, artificial intelligence, robotics, and algorithms (STARA): employees' perceptions of our future workplace. J. Manag. Organ..

[21] Guilmette M., Mulvihill K., Villemaire-Krajden R., Barker E.T. (2019). Past and present participation in extracurricular activities is associated with adaptive self-regulation of goals, academic success, and emotional wellbeing among university students. Learn. Individ. Differ..

[22] Luo M., Zhao L., Lyu B. (2022). Exploring the fuzzy integrated assessment of college students' education for innovative entrepreneurship under the background of Internet+. Secur. Commun. Networks.

[23] Ouyang F., Zheng L., Jiao P. (2022). Artificial intelligence in online higher education: a systematic review of empirical research from 2011 to 2020. Springer US.

[24] Li J., Che W. (2022). Challenges and coping strategies of online learning for college students in the context of COVID-19: a survey of Chinese universities. Sustain. Cities Soc..

[25] Qureshi M.A., Khaskheli A., Qureshi J.A., Raza S.A., Yousufi S.Q. (2021). Factors affecting students' learning performance through collaborative learning and engagement. Interact. Learn. Environ..

[26] Barth Vedøy I., Anderssen S.A., Tjomsland H.E., Skulberg K.R., Thurston M. (2020). Physical activity, mental health and academic achievement: a cross-sectional study of Norwegian adolescents. Ment. Health Phys. Act..

[27] Dekker I., De Jong E.M., Schippers M.C., De Bruijn-Smolders M., Alexiou A., Giesbers B. (2020). Optimizing students' mental health and academic performance: AI-enhanced life crafting. Front. Psychol..

[28] Abdullah S.I.N.W., Arokiyasamy K., Goh S.L., Culas A.J., Manaf N.M.A. (2022). University students' satisfaction and future outlook towards forced remote learning during a global pandemic. Smart Learn. Environ..

[29] Fui-Hoon Nah F., Zheng R., Cai J., Siau K., Chen L. (2023). Generative AI and ChatGPT: applications, challenges, and AI-human collaboration. J. Inf. Technol. Case Appl. Res..

[30] Boahene K.O., Fang J., Sampong F. (Apr. 2019). Social media usage and tertiary students' academic performance: examining the influences of academic self-efficacy and innovation characteristics. Sustain. Times.

[31] Alturki S., Cohausz L., Stuckenschmidt H. (2022). Predicting Master's students' academic performance: an empirical study in Germany. Smart Learn. Environ.

[32] Yu A.Y., Tian S.W., Vogel D., Chi-Wai Kwok R. (Dec. 2010). Can learning be virtually boosted? An investigation of online social networking impacts. Comput. Educ..

[33] Ali F., Nair P.K., Hussain K. (2016). An assessment of students' acceptance and usage of computer supported collaborative classrooms in hospitality and tourism schools. J. Hosp. Leis. Sport Tour. Educ..

[34] Jia S., Khassawneh O., Mohammad T., Cao Y. (2023). Knowledge-oriented leadership and project employee performance: the roles of organisational learning capabilities and absorptive capacity. Curr. Psychol..

[35] Naser S.A., Zaqout I., Ghosh M.A., Atallah R., Alajrami E. (2015). Predicting student performance using artificial neural network: in the faculty of engineering and information technology. Int. J. Hybrid Inf. Technol..

[36] Ali Z., Gongbing B., Mehreen A. (2018). Understanding and predicting academic performance through cloud computing adoption: a perspective of technology acceptance model. Springer Berlin Heidelberg.

[37] Kaliisa R., Rienties B., Mørch A.I., Kluge A. (2022). Social learning analytics in computer-supported collaborative learning environments: a systematic review of empirical studies. Comput. Educ. Open.

[38] Grover P., Kar A.K., Dwivedi Y.K. (2022).

[39] Hill J.R., Song L., West R.E. (2009). Social learning theory and web-based learning environments: a review of research and discussion of implications. Int. J. Phytoremediation.

[40] Ma X., Huo Y. (2023). Are users willing to embrace ChatGPT? Exploring the factors on the acceptance of chatbots from the perspective of AIDUA framework. Technol. Soc..

[41] Alamri M.M., Almaiah M.A., Al-Rahmi W.M. (2020). Social media applications affecting students' academic performance: a model developed for sustainability in higher education. Sustain. Times.

[42] Yu H. (2023). Reflection on whether Chat GPT should be banned by academia from the perspective of education and teaching. Front. Psychol..

[43] Zawacki-Richter O., Marín V.I., Bond M., Gouverneur F. (2019). Systematic review of research on artificial intelligence applications in higher education – where are the educators?. Int. J. Educ. Technol. High. Educ..

[44] Tsai M.L., Ong C.W., Chen C.L. (2023). Exploring the use of large language models (LLMs) in chemical engineering education: building core course problem models with Chat-GPT. Educ. Chem. Eng..

[45] Salas-Pilco S.Z., Yang Y. (2022). Artificial intelligence applications in Latin American higher education: a systematic review. Int. J. Educ. Technol. High. Educ..

[46] Pang H. (2023). Determining the influence of depressive mood and self-disclosure on problematic mobile app use and declined educational attainment: insight from stressor-strain-outcome perspective. Educ. Inf. Technol..

[47] Arunachalam A.S., Velmurugan T. (2018). Analyzing student performance using evolutionary artificial neural network algorithm. Int. J. Eng. Technol..

[48] Zacharis N.Z. (2016). Predicting student academic performance in blended learning using artificial neural networks. Int. J. Artif. Intell. Appl..

[49] Dawoodbhoy F.M. (2021). AI in patient flow: applications of artificial intelligence to improve patient flow in NHS acute mental health inpatient units. Heliyon.

[50] Javaid M., Haleem A., Pratap R. (2023). ChatGPT for healthcare services : an emerging stage for an innovative perspective. BenchCouncil Trans. Benchmarks, Stand. Eval.

[51] Lee E.E. (2021). Artificial intelligence for mental health care: clinical applications, barriers, facilitators, and artificial wisdom. Biol. Psychiatry Cogn. Neurosci. Neuroimaging.

[52] Cerasa A., Gaggioli A., Marino F., Riva G., Pioggia G. (2022). The promise of the metaverse in mental health: the new era of MEDverse. Heliyon.

[53] Rubeis G. (2022). iHealth: the ethics of artificial intelligence and big data in mental healthcare. Internet Interv.

[54] Theophilou E. (2023). Learning to prompt in the classroom to understand AI limits: a pilot study. Lect. Notes Comput. Sci..

[55] Liberatore M.J., Wagner W.P. (2021). Virtual, mixed, and augmented reality: a systematic review for immersive systems research. Virtual Real..

[56] Hee Lee D., Yoon S.N. (2021). Application of artificial intelligence-based technologies in the healthcare industry: opportunities and challenges. Int. J. Environ. Res. Public Health.

[57] Alam M.M.D., Alam M.Z., Rahman S.A., Taghizadeh S.K. (2021). Factors influencing mHealth adoption and its impact on mental well-being during COVID-19 pandemic: a SEM-ANN approach. J. Biomed. Inform..

[58] Wilson R.L. (2023). Artificial intelligence: an eye cast towards the mental health nursing horizon. Int. J. Ment. Health Nurs..

[59] Johnson A. (2020). A review and agenda for examining how technology-driven changes at work will impact workplace mental health and employee well-being. Aust. J. Manag..

[60] Giunchiglia F., Zeni M., Gobbi E., Bignotti E., Bison I. (2018). Mobile social media usage and academic performance. Comput. Human Behav..

[61] Shafiq M., Parveen K. (2023). Social media usage: analyzing its effect on academic performance and engagement of higher education students. Int. J. Educ. Dev..

[62] Alshuaibi M.S.I., Alshuaibi A.S.I., Shamsudin F.M., Arshad D.A. (2018). Use of social media, student engagement, and academic performance of business students in Malaysia. Int. J. Educ. Manag..

[63] Malik A., Dhir A., Kaur P., Johri A. (2021). Correlates of social media fatigue and academic performance decrement: a large cross-sectional study. Inf. Technol. People.

[64] Pang H. (2022). Connecting mobile social media with psychosocial well-being: understanding relationship between WeChat involvement, network characteristics, online capital and life satisfaction. Soc. Networks.

[65] Barton B.A., Adams K.S., Browne B.L., Arrastia-Chisholm M.C. (2021). The effects of social media usage on attention, motivation, and academic performance. Act. Learn. High. Educ..

[66] Pallavi Gupta C., Singh B., Marwaha T. (2013). Relationship between social media and academic performance in distance education. Univers. J. Educ. Res..

[67] Asante E., Martey E.M. (2015). Impact of social media usage on academic performance of tertiary institution students. J. Adv. Res. Bus. Manag. Account. (ISSN 2456-3544).

[68] Khan I.U., Hameed Z., Yu Y., Khan S.U. (Nov. 2017). Assessing the determinants of flow experience in the adoption of learning management systems: the moderating role of perceived institutional support. Behav. Inf. Technol..

[69] Annamalai N., Foroughi B., Iranmanesh M., Buathong S. (2020). Needs and Facebook addiction: how important are psychological well-being and performance-approach goals?. Curr. Psychol..

[70] Conway M., O'Connor D. (2016). Social media, big data, and mental health: current advances and ethical implications. Curr. Opin. Psychol..

[71] Xu S., Wang Z., David P. (2022). Social media multitasking (SMM) and well-being: existing evidence and future directions. Curr. Opin. Psychol..

[72] Ponnusamy S., Iranmanesh M., Foroughi B., Hyun S.S. (2020). Drivers and outcomes of Instagram addiction: psychological well-being as moderator. Comput. Human Behav..

[73] Kross E., Verduyn P., Sheppes G., Costello C.K., Jonides J., Ybarra O. (2021). Social media and well-being: pitfalls, progress, and next steps. Trends Cogn. Sci..

[74] Twenge J.M., Martin G.N. (2020). Gender differences in associations between digital media use and psychological well-being: evidence from three large datasets. J. Adolesc..

[75] Best P., Manktelow R., Taylor B. (2014). Online communication, social media and adolescent wellbeing: a systematic narrative review. Child. Youth Serv. Rev..

[76] Luqman A., Talwar S., Masood A., Dhir A. (2021). Does enterprise social media use promote employee creativity and well-being?. J. Bus. Res..

[77] Bano S., Cisheng W., Khan A.N., Khan N.A. (2019). WhatsApp use and student's psychological well-being: role of social capital and social integration. Child. Youth Serv. Rev..

[78] Fan X., Deng N., Dong X., Lin Y., Wang J. (2019). Do others' self-presentation on social media influence individual's subjective well-being? A moderated mediation model. Telemat. Informatics.

[79] Gerson J., Plagnol A.C., Corr P.J. (2016). Subjective well-being and social media use: do personality traits moderate the impact of social comparison on Facebook?. Comput. Human Behav..

[80] Bruggeman H., Van Hiel A., Van Hal G., Van Dongen S. (2019). Does the use of digital media affect psychological well-being? An empirical test among children aged 9 to 12. Comput. Human Behav..

[81] Hardy B.W., Castonguay J. (2018). The moderating role of age in the relationship between social media use and mental well-being: an analysis of the 2016 General Social Survey. Comput. Human Behav..

[82] Rasmussen E.E., Punyanunt-Carter N., LaFreniere J.R., Norman M.S., Kimball T.G. (2020). The serially mediated relationship between emerging adults' social media use and mental well-being. Comput. Human Behav..

[83] Coyne S.M., Rogers A.A., Zurcher J.D., Stockdale L., Booth M. (2020). Does time spent using social media impact mental health?: an eight year longitudinal study. Comput. Human Behav..

[84] Allal-Chérif O., Yela Aránega A., Castaño Sánchez R. (2021). Intelligent recruitment: how to identify, select, and retain talents from around the world using artificial intelligence. Technol. Forecast. Soc. Change.

[85] Muro A., Soler J., Cebolla À., Cladellas R. (2018). A positive psychological intervention for failing students: does it improve academic achievement and motivation? A pilot study. Learn. Motiv..

[86] Samaha M., Hawi N.S. (2016). Relationships among smartphone addiction, stress, academic performance, and satisfaction with life. Comput. Human Behav..

[87] Boer M., Stevens G.W.J.M., Finkenauer C., de Looze M.E., van den Eijnden R.J.J.M. (2021). Social media use intensity, social media use problems, and mental health among adolescents: investigating directionality and mediating processes. Comput. Human Behav..

[88] Embarak O.H. (2022). Internet of Behaviour (IoB)-based AI models for personalized smart education systems. Procedia Comput. Sci..

[89] Tomporowski P.D., Davis C.L., Miller P.H., Naglieri J.A. (2008). Exercise and children's intelligence, cognition, and academic achievement. Educ. Psychol. Rev..

[90] Zang J., Kim Y., Dong J. (2022). New evidence on technological acceptance model in preschool education: linking project-based learning (PBL), mental health, and semi-immersive virtual reality with learning performance. Front. Public Heal..

[91] Beerbaum Dr D.O. (2023). Generative artificial intelligence (GAI) with chat GPT for accounting – a business case. SSRN Electron. J..

[92] Kinshuk, Chen N.S., Cheng I.L., Chew S.W. (2016). Evolution is not enough: revolutionizing current learning environments to smart learning environments. Int. J. Artif. Intell. Educ..

[93] Criado J.I., Gil-Garcia J.R. (2019). Creating public value through smart technologies and strategies: from digital services to artificial intelligence and beyond. Int. J. Public Sect. Manag..

[94] Lu H., Li Y., Chen M., Kim H., Serikawa S. (2018). Brain intelligence: go beyond artificial intelligence. Mob. Networks Appl.

[95] Smart M., Felton J., Meghea C., Buchalski Z., Maschino L., Sadler R. (2021). Is a school's neighborhood physical disorder related to its academic outcomes?. Child Youth Care Forum.

[96] Al-Marghilani A. (2022). Artificial intelligence-enabled cyberbullying-free online social networks in smart cities. Int. J. Comput. Intell. Syst..

[97] Martinez-Perez C., Alvarez-Peregrina C., Villa-Collar C., Sánchez-Tena M.Á. (2020). Current state and future trends: a citation network analysis of the academic performance field. Int. J. Environ. Res. Public Health.

[98] Biswas S.S. (2023). Role of chat GPT in public health. Ann. Biomed. Eng..

[99] Lei L., Li J., Li W. (2023). Assessing the role of artificial intelligence in the mental healthcare of teachers and students. Soft Comput..

[100] Fonseca D., Martí N., Redondo E., Navarro I., Sánchez A. (2014). Relationship between student profile, tool use, participation, and academic performance with the use of Augmented Reality technology for visualized architecture models. Comput. Human Behav..

[101] Zhang X., Abbas J., Shahzad M.F., Shankar A., Ercisli S., Dobhal D.C. (2024). Association between social media use and students ’ academic performance through family bonding and collective learning : the moderating role of mental well-being. Educ. Inf. Technol..

[102] Hair J., Hollingsworth C.L., Randolph A.B., Chong A.Y.L. (2017). An updated and expanded assessment of PLS-SEM in information systems research. Ind. Manag. Data Syst..

[103] Martins J.M., Shahzad M.F., Javed I. (2023). Assessing the impact of workplace harassment on turnover intention : evidence from the banking industry.

[104] Gong Y. (2021). Application of virtual reality teaching method and artificial intelligence technology in digital media art creation. Ecol. Inform..

[105] Li K. (2023). Determinants of college students' actual use of AI-based systems: an extension of the technology acceptance model. Sustainability.

[106] Shoufan A. (2023). Exploring students' perceptions of ChatGPT: thematic analysis and follow-up survey. IEEE Access.

[107] Zhao L. (2023). Social media addiction and its impact on college students' academic performance: the mediating role of stress. Asia-Pacific Educ. Res..

[108] Popescu E. (2014). Providing collaborative learning support with social media in an integrated environment. World Wide Web.

[109] Hu Z., Qin J. (2018). Generalizability of causal inference in observational studies under retrospective convenience sampling. Stat. Med..

[110] Shahzad M.F., Xu S., Lim W.M., Hasnain M.F., Nusrat S. (2024). Cryptocurrency awareness, acceptance, and adoption: the role of trust as a cornerstone. Humanit. Soc. Sci. Commun.

[111] Roscoe A.M., Lang D., Sheth J.N. (1975). Follow-up methods, questionnaire length, and market differences in mail surveys. J. Mark..

[112] Ferber R. (1977). Research by convenience. J. Consum. Res..

[113] Shahzad M.F., Xu S., Rehman O., Javed I. (2023). Impact of gamification on green consumption behavior integrating technological awareness , motivation , enjoyment and virtual CSR. Sci. Rep..

[114] Farrukh M., Xu S., Baheer R., Ahmad W. (2023). Unveiling the role of supply chain parameters approved by blockchain technology towards firm performance through trust : the moderating role of government support. Heliyon.

[115] Farrukh M., Xu S., Naveed W., Nusrat S. (2023). Investigating the impact of artificial intelligence on human resource functions in the health sector of China : a mediated moderation model. Heliyon.

[116] Hair J.F., Ringle C.M., Sarstedt M. (2013). Partial least squares structural equation modeling: rigorous applications, better results and higher acceptance. Long Range Plann..

[117] Babin B.J., Grif M., H J.F. (2015).

[118] Podsakoff P.M., MacKenzie S.B., Lee J.Y., Podsakoff N.P. (2003). Common method biases in behavioral research: a critical review of the literature and recommended remedies. J. Appl. Psychol..

[119] Bagozzi R.P., Edwards J.R. (1998). Organ. Res. Methods.

[120] Fuller C.M., Simmering M.J., Atinc G., Atinc Y., Babin B.J. (2015). Common methods variance detection in business research. J. Bus. Res..

[121] Fornell C., Larcker D.F. (1981). Evaluating structural equation models with unobservable variables and measurement error. J. Mark. Res..

[122] Henseler J., Ringle C.M., Sarstedt M. (2015). A new criterion for assessing discriminant validity in variance-based structural equation modeling. J. Acad. Mark. Sci..

[123] Fassott G. (2010). Handbook of partial least squares. Handb. Partial Least Squares.

[124] Shahzad M.F., Xu S., Khan K.I., Hasnain M.F. (2023). Effect of social influence , environmental awareness , and safety affordance on actual use of 5G technologies among Chinese students. Sci. Rep..

[125] Shahzad M.F., Xu S., Baheer R. (2024).

[126] Sarstedt M., Ringle C.M., Hair J.F. (2022). Handbook of Market Research.

[127] Ali S., Yan Q., Sun H., Irfan M. (2023). Sustainable green revolution through the development of solar power projects in Pakistan: a techno-economic analysis. Environ. Sci. Pollut. Res..

[128] Bickman L. (2020). Improving mental health services: a 50-year journey from randomized experiments to artificial intelligence and precision mental health. Springer US.

[129] Crompton H., Burke D. (2023). Artificial intelligence in higher education: the state of the field. Int. J. Educ. Technol. High. Educ..

[130] Santoveña-Casal S. (2019). The impact of social media participation on academic performance in undergraduate and postgraduate students. Int. Rev. Res. Open Distance Learn..

[131] Oberiri A.D. (2019). The influence of social media on academic performance of taraba state university undergraduate students. Online J. Commun. Media Technol..

[132] Mousavi Baigi S.F., Sarbaz M., Ghaddaripouri K., Ghaddaripouri M., Mousavi A.S., Kimiafar K. (2023). Attitudes, knowledge, and skills towards artificial intelligence among healthcare students: a systematic review. Heal. Sci. Reports.

[133] Al-Menayes J.J. (2015). Social media use, engagement and addiction as predictors of academic performance. Int. J. Psychol. Stud..

[134] Mirzakhani K., Shoorab N.J., Akbari A., Khadivzadeh T. (2022). High-risk pregnant women's experiences of the receiving prenatal care in COVID-19 pandemic: a qualitative study. BMC Pregnancy Childbirth.

[135] Al-Rahmi W.M., Zeki A.M. (2017). A model of using social media for collaborative learning to enhance learners' performance on learning. J. King Saud Univ. - Comput. Inf. Sci..

[136] Scott H., Woods H.C. (2019). Understanding links between social media use, sleep and mental health: recent progress and current challenges. Curr. Sleep Med Reports.

[137] Young L., Kolubinski D.C., Frings D. (2020). Attachment style moderates the relationship between social media use and user mental health and wellbeing. Heliyon.

[138] Sadagheyani H.E., Tatari F. (2021). Investigating the role of social media on mental health. Ment. Heal. Soc. Incl..

[139] Bekalu M.A., McCloud R.F., Viswanath K. (2019). Association of social media use with social well-being, positive mental health, and self-rated health: disentangling routine use from emotional connection to use. Heal. Educ. Behav..

[140] Ulvi O., Karamehic-Muratovic A., Baghbanzadeh M., Bashir A., Smith J., Haque U. (2022). Social media use and mental health: a global analysis. Epidemiologia.

[141] Hilal A.M., Alfurhood B.S., Al-Wesabi F.N., Hamza M.A., Al Duhayyim M., Iskandar H.G. (2022). Artificial intelligence based sentiment analysis for health crisis management in smart cities. Comput. Mater. Contin..

[142] Ruthig J.C., Haynes T.L., Stupnisky R.H., Perry R.P. (2009). Perceived academic control: mediating the effects of optimism and social support on college students' psychological health. Soc. Psychol. Educ..

[143] Clarke T. (2020). Children's wellbeing and their academic achievement: the dangerous discourse of ‘trade-offs’ in education. Theory Res. Educ..

[144] Graham S. (2019). Artificial intelligence for mental health and mental illnesses: an overview. Curr. Psychiatry Rep..

[145] Richards D., Caldwell P.H.Y., Go H. (Dec. 2015). Impact of social media on the health of children and young people. J. Paediatr. Child Health.

