# Supplementary material for: Artificial intelligence and social media on academic performance and mental well-being: Student perceptions of positive impact in the age of smart learning
Source: Heliyon. 2024 Apr 15;10(8):e29523. doi: 10.1016/j.heliyon.2024.e29523 (PMC11043955; doi:10.1016/j.heliyon.2024.e29523)
Supplement: Multimedia component 1 [file mmc1.docx]

**Artificial intelligence, ChatGPT and use of social media by Chinese students and their impact on their academic and mental health**

**Note:** I am conducting research as a student of PhD in Management Sciences and Engineering School of Economics and Management, Beijing University of Technology, Beijing China. I am conducting research on **“Artificial intelligence, ChatGPT and use of social media by Chinese students and their impact on their academic and mental health”.** You are requested to spare your precious time to complete this survey. Your specific answers will be completely anonymous & confidential, but your views, in combination with those of others, are extremely important. Your cooperation is highly appreciated. Thanks once again for your time and cooperation.

**Section-1 Personal Memoranda**

1. Name (Optional)------------------------------------------------------------------------
2. Phone No (Optional)-------------------------------------------------------------------
3. Email Address (Optional)---------------------------------------------------------------
4. Designation-------------------------------------------------------------------------------
5. Department--------------------------------------------------------------------------------
6. Experience of working--------------------------------------------------------------------
7. Qualification:

- Intermediate -----------------------------------------------------------------------
- Bachelor --------------------------------------------------------------
- Master-----------------------------------------------------------------------------
- Ph.D.----------------------------------------------------------------------------

1. Gender of Respondent:

- Male------------------------------------
- Female---------------------------------

1. Age

- 18-24
- 24-30
- 30-36

1. Daily internet use

- 1-4 hours
- 4-8 hours
- More than 8 hours

**Section – 2 (Please reply all the research questions.)**

1. **Artificial Intelligence**

| **Encircle only one number from 1-5 that indicates your disagreement or agreement** | | **Strongly**  **disagree** | **Disagree** | **Neutral** | **Agree** | **Strongly agree** |
| --- | --- | --- | --- | --- | --- | --- |
| AI1 | I intended to adopt AI-based systems for my study. |  |  |  |  |  |
| AI2 | I think that AI-based systems help me improve my study performance. |  |  |  |  |  |
| AI3 | I think designing AI-based systems helps me improve my learning performance. |  |  |  |  |  |
| AI4 | I believe that AI-based systems help me cooperate well with others. |  |  |  |  |  |
| AI5 | I believe that AI-based systems help me enhance my learning efficient. |  |  |  |  |  |
| AI6 | I am amazed by the capabilities of ChatGPT. |  |  |  |  |  |
| AI7 | ChatGPT is a helpful and effective technology for learning. |  |  |  |  |  |
| AI8 | Asking follow-up questions help to find the correct answer. |  |  |  |  |  |

1. **Social Media**

| **Encircle only one number from 1-5 that indicates your disagreement or agreement** | | **Strongly**  **disagree** | **Disagree** | **Neutral** | **Agree** | **Strongly agree** |
| --- | --- | --- | --- | --- | --- | --- |
| SM1 | I feel a sense of community learning becomes interactive using social media. |  |  |  |  |  |
| SM2 | I can get faster feedback from my peers using social media. |  |  |  |  |  |
| SM3 | I can get faster feedback from my instructor using social media. |  |  |  |  |  |
| SM4 | I increase my class participation when I can contribute through social media. |  |  |  |  |  |
| SM5 | I multitask with my social media account while studying. |  |  |  |  |  |
| SM6 | I can communicate effectively using social media. |  |  |  |  |  |

1. **Academic Performance**

| **Encircle only one number from 1-5 that indicates your disagreement or agreement** | | **Strongly**  **disagree** | **Disagree** | **Neutral** | **Agree** | **Strongly agree** |
| --- | --- | --- | --- | --- | --- | --- |
| AP1 | I focus on the quality of the study using AI and social media. |  |  |  |  |  |
| AP2 | I focus on the study's accuracy through AI and social media. |  |  |  |  |  |
| AP3 | I have obtained personal career goals. |  |  |  |  |  |
| AP4 | I have developed the skills needed for my future career using AI and social media. |  |  |  |  |  |
| AP5 | Continuous seeking out career development opportunities using new applications etc. ChatGPT. |  |  |  |  |  |

1. **Mental Well-being**

| **Encircle only one number from 1-5 that indicates your disagreement or agreement** | | **Strongly**  **disagree** | **Disagree** | **Neutral** | **Agree** | **Strongly agree** |
| --- | --- | --- | --- | --- | --- | --- |
| MWB1 | I have been feeling optimistic about the future. |  |  |  |  |  |
| MWB2 | I have been feeling relaxed. |  |  |  |  |  |
| MWB3 | I have been dealing with problems well. |  |  |  |  |  |
| MWB4 | I have been thinking clearly. |  |  |  |  |  |
| MWB5 | I have been feeling close to other people. |  |  |  |  |  |
| MWB6 | I have been able to make up my mind about things. |  |  |  |  |  |

1. **Smart Learning**

| **Encircle only one number from 1-5 that indicates your disagreement or agreement** | | **Strongly**  **disagree** | **Disagree** | **Neutral** | **Agree** | **Strongly agree** |
| --- | --- | --- | --- | --- | --- | --- |
| SM1 | I like to read diverse topics. |  |  |  |  |  |
| SM2 | I expect myself to support the group’s goals. |  |  |  |  |  |
| SM3 | I feel very good when I know I have outperformed other students. |  |  |  |  |  |
| SM4 | I enjoy learning about new topics and tools like Generated AI (ChatGPT). |  |  |  |  |  |
| SM5 | I find pleasure in learning new social media applications. |  |  |  |  |  |
